# Status one year after fertility assessment and counselling in women of reproductive age—a qualitative study

**DOI:** 10.1080/03009734.2018.1546243

**Published:** 2018-12-12

**Authors:** Randi Sylvest, Emily Koert, Ida Vittrup, Kathrine Birch Petersen, Anders Nyboe Andersen, Anja Pinborg, Lone Schmidt

**Affiliations:** a Department of Obstetrics and Gynecology, Fertility Clinic, Hvidovre Hospital, University of Copenhagen, Hvidovre, Denmark;; b Fertility Clinic, University Hospital Copenhagen, Rigshospitalet, Copenhagen, Denmark;; c Fertility Clinic, University Hospital of Zealand, Køge, Denmark;; d Department of Public Health, University of Copenhagen, Copenhagen, Denmark

**Keywords:** Childbearing, fertility, fertility assessment and counselling, postponing childbearing, qualitative interviews, women

## Abstract

**Introduction**: Over the past 50 years women and men have postponed family formation in high-income societies. Fertility assessment and counselling has been suggested as a method to reduce delayed childbearing and its consequences. This study explored women’s perceptions of how attending a fertility assessment intervention influenced their decisions and choices regarding family formation and childbearing.

**Material and methods**: Follow-up data from a longitudinal semi-structured qualitative interview study including 20 women aged 35–40 years seeking individual fertility counselling at the Fertility Assessment and Counselling Clinic at Rigshospitalet, Copenhagen, Denmark. The interviews were conducted one year after their consultation. Data were analysed by qualitative content analysis.

**Results**: The women perceived an increase in their knowledge after they had attended the counselling. The women saw the counselling as a catalyst for change—they changed their behaviour and relationship status. The women stopped thinking about the pros and cons of childbearing and acted instead. The women did not experience any regrets about acting. Some of the women felt that they were still in limbo as they were still in doubt concerning childbearing. The consultation had not given them an answer with a clear deadline in terms of delaying attempts to become pregnant, and this frustrated them.

**Conclusions**: Our study highlights the impact of a fertility assessment and counselling intervention which included a perceived increase in knowledge. The clinic allows for an individualized approach to fertility awareness which is necessary given the unique nature of childbearing decisions.

## Introduction

Men and women from high-income societies are postponing parenthood. As a result, the average age at first birth has been steadily increasing over the past decades. In Denmark, the postponement started 50 years ago, and the current average age at first birth for women is 29.2 years, whereas in 1987 it was 25.8 years (1). While there are benefits of delaying parenthood (e.g. financial stability, higher family functioning, and stability) (2), there are risks related to advanced maternal age such as increased maternal, fetal, and infant risks, age-related infertility, smaller family sizes than intended, and unintentional permanent childlessness (3,4), and women tend to overestimate the success rates of fertility treatment (3).

A large body of international literature confirms that men and women have significant knowledge gaps in their understanding of age-related fertility decline and the risks of delayed childbearing (e.g. 5–8), suggesting that they may be making decisions to postpone parenthood based on inaccurate knowledge. Fertility education and awareness has been suggested as a method of increasing knowledge and reducing the negative consequences of delayed childbearing. There is a need to investigate the impact of this type of intervention to see if it influences choices on the critical issues of delayed childbearing and gaps in fertility awareness and knowledge.

A small but growing number of researchers have developed fertility education and awareness interventions and tested their efficacy. These include educational websites (e.g. YourFertility.au.org (9); MyFertilityChoices.com (10)), educational brochures/pamphlets/slideshows (e.g. 11–14), and an educational video (15). The focus in these educational efforts has been on increasing knowledge and influencing changes in fertility intentions (e.g. preference for timing of childbearing) using standardized interventions. These studies generally used a pre–post design to measure changes in knowledge levels and intentions. Only the study by Daniluk and Koert included a follow-up period of six months after exposure to an educational website. The researchers found that in their sample of 199 childbearing-aged women and men the increase in knowledge levels immediately post intervention largely returned to baseline by follow-up (10). Very few interventions offer tailored information according to individual risk factors. The Reproductive Life Plan (RLP) (16,17) is a counselling tool that helps men and women reflect on their intentions and strategies for family planning within their personal life circumstances. This intervention has been found to increase fertility knowledge and shows promise as a tool to promote reproductive health (16–18). The FertiSTAT (19) is an online fertility awareness tool that provides tailored fertility guidance directly to the user based on personal risk factors for fertility. This intervention has been found to be effective in identifying women with risks for infertility (19). However, our knowledge of the impact of tailored fertility awareness interventions on future childbearing decision-making and family formation is limited.

In Denmark, the Fertility Assessment and Counselling (FAC) Clinic at Rigshospitalet, Copenhagen, was developed as a personalized fertility awareness intervention for men and women to provide individual assessment and guidance related to their personal fertility levels. This is the only tailored intervention that provides personal guidance based on reproductive history and risk factors in addition to endocrine and detailed sonographic assessments of ovarian reserve (i.e. anti-Müllerian hormone test and antral follicle count). Women and men are self-referred and receive counselling regarding their fertility risk factors and ovarian reserve or sperm quality (20). Various studies testing this concept have been published (21,22), including a qualitative study exploring attitudes towards family formation in 20 women (10 cohabitating, 10 single) attending the FAC Clinic (23). The current study interviewed the same sample, one year after the intervention.

The purpose of the study was to explore women’s perceptions of how attending a fertility assessment intervention influenced their decisions and choices regarding family formation and childbearing.

## Material and methods

Follow-up data were collected from a longitudinal semi-structured qualitative interview study including 20 women aged 35–40 years seeking individual fertility counselling. The interviews were conducted one year after their consultation at the FAC Clinic in Copenhagen, Denmark. The FAC Clinic offers women and men with no known fertility problems assessment and counselling regarding their present and future fertility. The clinic is state-funded and offers consultations free of charge, and men and women do not need a referral but can schedule an appointment by themselves. Clients fill out a questionnaire including items regarding socio-demographic background, reproductive and medical history, lifestyle, and behavioural exposures (for more detail, see 20). Men have a semen analysis, and women undergo a pelvic sonography for an antral follicle count (AFC) and provide a blood sample for measurement of anti-Müllerian hormone (AMH). Both tests measure a woman’s ovarian reserve, or the number of remaining oocytes (24). The pros and cons of AMH screening have been thoroughly discussed (24) and were found to meet the World Health Organization’s criteria (25) for an adequate screening test.

Study participants were 5 single and 15 cohabiting women, residents in the Capital Region of Copenhagen, Denmark. All had attended the FAC Clinic one year previously and had been interviewed immediately afterwards (23). In that interview, all participants agreed to be contacted to participate in a follow-up study one year after attending the FAC Clinic. For the follow-up study, the participants were first sent an email that included information about the follow-up study (e.g. the focus of the interview, time commitment, the interviewer’s training, and participation in the first study) and their right to decline to participate. Those who did not reply by email were contacted by phone to confirm that they had received the email and to answer any questions about the study. All contact was initiated by the first author. All 20 women who had participated in the first study (23) participated in the follow-up study. The participants were informed that they could withdraw from the study at any time.

We developed a semi-structured interview guide with open-ended questions focusing on their perceptions of the impact of the consultation at the FAC Clinic on their decisions regarding family formation, childbearing, and relationships in the last year. The questions included: What has happened the last year? What are your current thoughts about having a family? Why did you choose to go to the FAC Clinic last year? What have you gained from attending the FAC Clinic? What would you say to yourself if you met yourself one year earlier? What, if anything, would you do differently?

Depending on the participant’s preference, the interview took place in her own home, at her workplace, or at the FAC Clinic. All women were interviewed individually. The interviews were conducted by the first author (R.S.), a Master in Public Health Science specializing in qualitative methods. The interviews were audio-taped and transcribed verbatim. Transcripts were anonymized. The duration of the interviews varied from 20 to 90 min, with a mean time of 36 min. Transcripts were analysed according to qualitative content analysis (26). In order to ensure trustworthiness of data analysis, Lincoln and Guba’s guidelines (27) and the consolidated criteria for reporting qualitative research (COREQ) were followed (28). The transcripts were first read in detail to gain a broad idea of the content. Using an inductive process, meaning units of text (i.e. salient words, sentences, and paragraphs) were identified in the transcripts. Next, the meaning units were condensed and labelled with a code that reflected their meaning. Codes were grouped into subthemes, and subthemes were grouped into a theme, with the goal of capturing similarities and differences between the women’s experiences. Data were triangulated between three of the co-authors through ongoing discussions during the analysis process until consensus was reached. The remaining co-authors reviewed and confirmed the description of the themes. An example of the analytic scheme is included in Table 1. Selected quotations are presented in the text to illustrate the meaning of the theme.

**Table 1.** Sample analytic scheme.

Code	Subcode	Subtheme	Overall theme
Left boyfriend	Relationship change	Catalyst for change	Knowledge increased
Started fertility treatment	Behaviour change	Catalyst for change	
Pursued solo parenthood	Behaviour change	Catalyst for change	

### Ethical approval

The study followed the principles of the Declaration of Helsinki II for Medical Research. Written informed consent was obtained from all participants. Interviews were anonymized, and sensitive data were kept in a separate document. The Danish Data Protection Agency approved the study (SUND-2018–15). According to Danish legislation, interview studies do not require permission from the Scientific Ethical Committee.

## Results

General information on the participants’ characteristics is presented in Table 2. Since their consultation at the FAC Clinic, seven had started fertility treatment (with their partner or as a future solo mother), two had left their partner, and three had delivered a baby.

**Table 2.** Characteristics of the study population.

	Cohabiting /Single	Age (y)	Vocational training^a^	Income^b^	Duration of current relationship (y)	Starting fertility treatment	Currently pregnant/having children
Caitlin	Cohabiting	36	Long	Low	5	Yes	No
Caja	Cohabiting	36	Long	High	1	No	No
Callie	Cohabiting	35	Long	High	13	No	No
Camelia	Cohabiting	37	Long	High	3	No	Yes
Camma	Cohabiting	38	Long	High	5	No	Yes
Carmen	Cohabiting	39	Long	High	9	No	No
Caroline	Cohabiting	35	Long	Low	5	Yes	No
Catherine	Cohabiting	38	Long	Medium	2	No	No
Cecilie	Cohabiting	35	Medium	High	6	Yes	Yes
Cindy	Cohabiting	40	Medium	Medium	3	Yes	No
Sabina	Single	36	Long	Medium	–	No	No
Sally	Single	39	Long	Medium	–	Yes	No
Sandra	Cohabiting	40	Long	High	1	No	No
Sanne	Cohabiting	38	Long	Low	1	No	No
Sarah	Single	39	Long	Medium	–	Yes	No
Selma	Single	36	Short	Medium	–	No	No
Serena	Cohabiting	36	Medium	Medium	1	No	No
Shana	Cohabiting	38	Long	Medium	1	Yes	No
Signe	Cohabiting	36	Long	Low	1	No	No
Susie	Single	39	Long	Medium	–	No	No

a Long: 4 years or more of vocational training; Medium: 2–3 years of vocational training; Short: 1 year or less of vocational training.

b High: >134,000 Euros; Medium: 40,000–134,000 Euros; Low: 27,000–40,000 Euros..

The results represent 20 different women with 20 different stories. The overall theme was: ‘Knowledge increased’. The women shared how they had increased their knowledge on fertility/fertility-related issues after they had attended the counselling. In particular, the women felt they gained new knowledge about age-related fertility decline. The increased knowledge was viewed positively because it helped them feel better equipped to make informed decisions about childbearing. In some cases, this new knowledge caused them to start to try to conceive and/or pursue fertility treatment. ‘Catalyst for change’, ‘Staying in limbo’, and ‘Peace of mind’ were subthemes. The women could be represented in more than one subtheme.

The women experienced different reactions to similar results and made different choices depending on their life circumstances and readiness for parenthood. For example, on hearing they had ‘plenty of time’ to conceive, some women experienced peace of mind that they could continue to delay, whereas others became frustrated that the result did not force them to act.

### Catalyst for change

Some women described the counselling as a catalyst for change in their lives. Changes were made regarding their behaviour, relationship, or emotional and cognitive state. These changes were viewed positively. The women did not experience any regrets about acting.

Women described how the reality of their closing fertile window pushed them to make a decision rather than continuing to ruminate on the pros and cons of starting to conceive. Emotional readiness followed biological readiness.

I sent it off and let my body run ahead and my head will follow later. (Sandra)

The counselling was a reality check and cue to act, or as one woman vividly described:

It felt like my brain hit the wall. It was the only time in my life where I jumped before planning things. (Caja)

After the counselling, seven women started fertility treatment (with their partner or as a future solo mother). One year after the counselling, three had delivered a baby. Two of the seven started treatment as a solo mother, and five started with their partner. They sought treatment because they felt they could not wait to pursue parenthood any longer. They wanted to take action and fulfil their desire to have a child before it was too late. In the case of the two single women, this included deciding to pursue parenthood on their own while there was still a chance for a pregnancy, despite their desire for a partner and wish for a nuclear family. The single women hoped that they would find a partner in the future. Regardless of their life circumstances, the women who pursued treatment explained it was a relief to ‘do something’.

It was a relief when I found out that I had many eggs in spite of my high age, and it was a really good catalyst to seek a referral and to start treatment. (Sally)

Other women identified the counselling as a catalyst for change in their relationship. For example, the counselling facilitated conversations with their partner about childbearing:

It has definitely been useful because it has both been the entrance to a conversation and started some things and emphasized an important part of doing something about it and it has supported me in what I want and brought him into it. (Caroline)

It also motivated the women to ‘put their foot down’ and give their partner an ultimatum about parenthood or, in two cases, to leave their partners to increase their chance of becoming a parent:

I put my foot down and stated my position and I think it occurred to him that I meant it seriously, now or never, or I would leave him. (Callie)

Finally, a couple of the women described changing their lifestyle behaviours (e.g. reducing alcohol intake and/or cigarette smoking) in preparation for a future pregnancy, after learning about health and lifestyle-related risks to fertility in the counselling.

### Staying in limbo

Some women felt that the counselling left them in ‘limbo’ and doubt regarding childbearing. Being in limbo was experienced negatively, and frustration was the most common feeling. One year after the counselling, some of the women were still in this ‘limbo’ state, particularly in relation to their decision-making about parenthood and their relationship.

The women attended the counselling seeking concrete answers about their fertility status and how long they could safely delay childbearing. They felt frustrated when they were not given a clear and exact deadline.

[I received] Irritatingly few answers actually because I was told that ‘It [my fertility] does not seem to be a problem at all’, where I might had expected to receive a deadline that if you want this, then you need to do it within a certain period of time. (Serena)

Being in ‘limbo’ was also frustrating because they felt stuck between acting and not acting:

We just have to make some kind of decision, but we can’t. So it’s such an evil limbo, where you almost hope that time expires, because then there will be some closure. (Carmen)

In addition to experiencing frustration with the result of the counselling, the women were also unsatisfied with themselves for remaining in limbo and failing to act. For example, one participant described that despite knowing she should leave her relationship to find a partner willing to become a parent, she continued to stay.

I talk the talk—but I can’t walk the walk—I just stay with him. (Caroline)

Others, in particular the single women, felt lost and uncertain due to the dilemma of choosing between two non-ideal options:

The choice is between having no children or to have children where one has to compromise on some things either in terms of parenting alone or choosing a man who may not be 100% the man one hoped for or expected. (Sabina)

This dilemma caused some of the single women to stay ‘in limbo’ and not take action regarding their fertility. Staying in limbo was stressful for those who felt ready to become a parent but were waiting for their circumstances (i.e. to find a partner) to change before pursuing parenthood.

### Peace of mind

Several women described how the counselling gave them peace of mind in regard to their decision-making about childbearing. Peace of mind was seen as a positive outcome of the counselling, in particular because they were given time and felt less pressure to decide or act.

Some felt peace of mind that their fertility was ‘normal’ allowing them some time before they needed to conceive.

The consultation gave me peace of mind because I could understand that my fertility was normal, or in other words, good. (Sabina)

They were relieved that they did not have to act (i.e. try to conceive) right now.

The answers allowed me to postpone the decision for some time because they were more positive for the both of us than I expected. (Catherine)

Others felt they had more time and less pressure to have conversations with their partner regarding childbearing.

I felt more calm and relaxed in relation to when we would need to have the talk. (Serena)

Others felt peace of mind that they had made an informed, realistic decision with the knowledge they felt they gained by attending the counselling.

It gave me some answers to some questions that helped me make some more informed choices. (Susie)

For some, their peace of mind increased in the year since the counselling. They felt more settled, calm, and less panicked about making a decision.

A year ago I felt more panicked than I really do now. (Sabina)

One of the women felt less stressed even though she was one year older and her biological clock was ticking.

I feel less stressed now about having children than I was a year ago, although it really should be reversed. (Caja)

## Discussion

In contrast to other fertility awareness interventions that provide the same information to everyone (e.g. brochures and online educational strategies), the FAC Clinic focuses on each individual and provides personalized information and guidance regarding fertility. The findings of this study suggest that the individualized, personalized approach is a promising intervention to increase fertility awareness and impact childbearing decision-making. In this approach, the staff focuses on the individual’s current life circumstances and can alter the feedback and provision of information based on the results of the individual’s fertility tests and her questions and concerns. The women in the study perceived the environment as safe, respectful, and non-judgemental and felt they could be open and honest.

Very few fertility awareness interventions offer tailored guidance according to individual risk factors. Research shows the value in this type of fertility awareness tool (16–19). However, we do not know about the long-term impact of these interventions on childbearing decision-making and family formation preferences. Our study is the first to test a fertility awareness intervention that includes tailored guidance based on reproductive history and risk factors along with tests of ovarian reserve (AMH test and AFC count) and includes a follow-up period of one year. Our findings demonstrate that attending the FAC Clinic was a catalyst for change, not only concerning childbearing but also regarding the women’s relationship. One year later, the women described 20 unique stories. These findings underscore the individualized nature of women’s childbearing decision-making and family formation preferences and suggest a need to focus on the individual in fertility awareness strategies, in order to provide relevant and useful information.

Previous research has highlighted that, for many women, delayed childbearing is ‘rarely a conscious choice’ and that they do not feel they have ultimate control regarding timing of childbearing, often due to their personal, relational, and economic circumstances (29, p. 30). In contrast, this study’s findings suggest that regardless of their life circumstances, attending the fertility counselling felt like an active action to seek out information about their fertility, which was a relief to some who felt uncertain about how long they could delay childbearing.

The findings suggest that the knowledge the women perceived they gained through the fertility counselling served as what the health belief model (30,31) calls a ‘cue to action’. For some, it was the catalyst for making changes in their relationship (i.e. leaving a partner) or fertility behaviour (i.e. starting to conceive, seeking fertility treatment) or lifestyle behaviours (i.e. reducing alcohol intake or smoking), while, for others, it was deciding to wait to conceive with the peace of mind they had some time to do so. These women believed that the fertility counselling had provided them with a clear sense of their personal susceptibility to fertility problems and a better understanding of age-related fertility decline. This new knowledge was viewed positively because it helped them feel better equipped to make informed decisions related to childbearing and family formation. Those who remained in limbo were often those who perceived they had not received an accurate and well-defined answer regarding their fertility (i.e. being told they could likely safely wait a few years before trying to conceive). After the fertility counselling they did not feel particularly susceptible to fertility problems, and as such they did not experience a cue to action.

Interestingly, the same feedback (i.e. being told they could likely safely wait a few years to conceive) was perceived as a cue to action for some but not for others who felt dissatisfied and remained in limbo. Future research is needed to continue to understand what makes the difference in order to be the most effective in promoting satisfying childbearing decision-making. While a large number of the women reported feeling in limbo at the time of the fertility counselling in a previous study (23), one year later many had moved on and no longer felt this way. Future research is needed to examine the long-term impact of the intervention beyond the year mark.

### Strengths and limitations

Informed by Lincoln and Guba’s guidelines (27) and the COREQ criteria (28), several strategies were integrated into the study design in order to ensure trustworthiness of the analytic process and findings. Briefly, that included recruiting participants until data saturation was reached (in the first study), discussing the analysis and interpretation of the data over several time points with the co-authors from different disciplines, documenting the analytic process, and providing a description of the women who participated in the study so readers may judge transferability of the findings. The study participants had all chosen to seek individual fertility counselling, and we only included women of advanced reproductive age over 35 years. Hence, the results may not be directly transferred to the general population in regard to attitudes towards family formation and concerns of reproductive lifespan. Future research is needed to examine the impact of the intervention on women under 35.

## Conclusion

Our study highlights women’s perceptions of the impact of a personalized fertility assessment and counselling intervention which included experiencing an increase in knowledge and an impact on childbearing decision-making. The fertility assessment and counselling clinic provided an individualized approach that was frequently a catalyst for change concerning childbearing decisions and behaviour and is not available in standardized fertility awareness interventions. The women’s subsequent decisions depended on their current life circumstances, fertility status, and readiness for parenthood. Additional longitudinal research is needed to test the effectiveness of the intervention on decision-making regarding family formation over a longer period of time and with larger samples of women of reproductive age.
